# The Fusion Oncogene FUS-CHOP Drives Sarcomagenesis of High-Grade Spindle Cell Sarcomas in Mice

**DOI:** 10.1155/2019/1340261

**Published:** 2019-07-25

**Authors:** Mark Chen, Eric S. Xu, Nathan H. Leisenring, Diana M. Cardona, Lixia Luo, Yan Ma, Andrea Ventura, David G. Kirsch

**Affiliations:** ^1^Department of Pharmacology and Cancer Biology, Duke University School of Medicine, Durham, NC 27708, USA; ^2^Medical Scientist Training Program, Duke University School of Medicine, Durham, NC 27708, USA; ^3^Department of Radiation Oncology, Duke University Medical Center, Durham, NC 27708, USA; ^4^Department of Pathology, Duke University Medical Center, Durham, NC 27708, USA; ^5^Cancer Biology and Genetics Program, Memorial Sloan Kettering Cancer Center, New York, NY 10065, USA

## Abstract

Myxoid liposarcoma is a malignant soft tissue sarcoma characterized by a pathognomonic t(12;16)(q13;p11) translocation that produces a fusion oncoprotein, FUS-CHOP. This cancer is remarkably sensitive to radiotherapy and exhibits a unique pattern of extrapulmonary metastasis. Here, we report the generation and characterization of a spatially and temporally restricted mouse model of sarcoma driven by FUS-CHOP. Using different Cre drivers in the adipocyte lineage, we initiated *in vivo* tumorigenesis by expressing FUS-CHOP in *Prrx1*+ mesenchymal progenitor cells. In contrast, expression of FUS-CHOP in more differentiated cells does not form tumors *in vivo*, and early expression of the oncoprotein during embryogenesis is lethal. We also employ *in vivo* electroporation and CRISPR technology to rapidly generate spatially and temporally restricted mouse models of high-grade FUS-CHOP-driven sarcomas for preclinical studies.

## 1. Introduction

Soft tissue sarcomas are tumors of the connective tissue that can arise anywhere in the body and are fatal in nearly 1/3 of patients. Myxoid liposarcoma (MLPS) is a malignant liposarcoma that accounts for approximately 30% of all liposarcomas, the most common soft tissue sarcoma subtype. Clinically, MLPS is distinguished by its remarkable response to radiation therapy compared to most other soft tissue sarcoma subtypes [1, 2]. Additionally, these tumors metastasize to bone, liver, and other soft tissue sites, whereas most other soft tissue sarcomas most commonly metastasize to the lung. MLPS occurs most often in the extremities, specifically within the thigh musculature [3].

The genetic hallmark of MLPS is the t(12;16)(q13;p11) translocation that is present in nearly 95% of cases [4, 5] and generates a novel fusion protein, FUS-CHOP [6]. The *TERT* promoter is also mutated in nearly 80% of MLPS, suggesting that the activation of telomere maintenance is important in MLPS tumorigenesis [7]. Exome analysis of MLPS has revealed few other recurrent mutations in coding sequences compared to other solid tumors [8]. These data suggest that the *FUS-CHOP* translocation is the predominant driver in MLPS tumorigenesis.

In the FUS-CHOP fusion oncoprotein, the N-terminus of FUS is joined via a unique linker region with the entire CHOP protein. Interestingly, experiments in transgenic mice showed that expression of the truncated form of FUS in the presence of aberrant CHOP was sufficient to generate tumors [9]. However, neither truncated FUS nor aberrant CHOP alone was sufficient for tumorigenesis. These experiments suggest a synergistic mechanism of tumorigenesis resulting from abnormal FUS and CHOP expression in MLPS. CHOP (also known as DDIT3/GADD153) is a C/EBP family protein that plays a role in adipogenesis and is induced by ER stress, growth arrest, and DNA damage [10–12]. Despite the roles of CHOP in cellular stress responses, it is not induced by ionizing radiation [13]. *FUS* homozygous knockout mice are radiosensitive and exhibit genomic instability [14, 15]. FUS, *FUsed in Sarcoma*, is an RNA-binding protein (RBP) that is a member of the FUS/EWS/TAF15 (FET) protein family of RBPs containing prion-like domains (PrLDs) [16, 17]. Other FET family members are commonly translocated in sarcoma and may transform mesenchymal progenitor cells that normally differentiate into bone, fat, cartilage, and muscle cells. At least four *FUS-CHOP* transcript variants exist, but the transcript structure does not affect prognosis [4]. Therefore, *FUS-CHOP* appears to be the primary oncogenic driver in MLPS, but the precise mechanism of MLPS tumorigenesis remains to be elucidated.

A critical question in MLPS biology is the identity of the cell of origin. It is possible that oncogenic fusions only transform cells if expressed in the appropriate cell type at a permissive stage of development. In this scenario, the expression of the oncogenic fusion in a different cell type or during the wrong developmental stage will not lead to transformation. In MLPS, the cell of origin is undefined. Previous mouse models of MLPS have expressed FUS-CHOP ubiquitously. While these studies reported generation of liposarcomas in mice, the tumors do not resemble human myxoid liposarcomas histologically [18, 19]. To study the cell of origin of myxoid liposarcoma, we generated novel genetically engineered mice with a Cre-activatable FUS-CHOP translocation transcript. We crossed these mice to lines that express Cre recombinase in mesenchymal and adipose tissue lineages because MLPS arises most commonly in the thigh and is likely of mesenchymal origin [20–23]. Finally, to more accurately model human disease with a translocation expressed from the endogenous *FUS* promoter, we attempted to use CRISPR technology for genome editing to generate the FUS-CHOP translocation at the endogenous mouse genes.

## 2. Materials and Methods

### 2.1. Mouse Model Generation and Animal Use

*Rosa26* LSL-FUS-CHOP/+ mice were generated by taking the human 7-2 FUS-CHOP translocation variant cDNA from the NCBI database and generating a targeting vector for the *Rosa26* locus [6]. The design of the targeting vector with a neomycin cassette flanked by *attB* and *attP* sites with a floxed transcription/translation “STOP” cassette upstream of FUS-CHOP is described further in Section 3. R1 mouse embryonic stem cells (ESCs) were electroporated using standard techniques, and ESCs were selected with neomycin. ESC clones were tested for successful targeting of the vector into the *Rosa26* locus and confirmed via PCR. Upon successful founder line generation, mice were crossed with *Rosa26* PhiC31/+ mice (JAX stock no. 007670 [24]) to recombine the *attB* and *attP* sites and delete the Neo cassette. Then, the mice were crossed with wild-type 129/SvJ mice to generate *Rosa26* LSL-FUS-CHOP/+ mice. These mice were crossed with the following cell-type-specific Cre and CreER lines: *Meox2-Cre* (JAX stock no. 003755 [25]), *PdgfRα-Cre* (JAX stock no. 013148 [26]), *Prrx1-Cre* (JAX stock no. 005584 [27]), *Prrx1-CreER-GFP* (JAX stock no. 029211 [28]), and *aP2-CreER* (a gift from the Yann Herault lab [29]).

### 2.2. In Vivo Cre Delivery or Activation

Mice lacking Cre expression were injected with an adenovirus expressing Cre, Ad5CMVCre (University of Iowa Viral Vector Core, VVC-U of Iowa-5), to activate recombination via Cre recombinase. Virus was prepared by mixing 25 *μ*L of Ad-Cre with 600 *μ*L minimal essential medium (Sigma-Aldrich, M4655). 3 *μ*L of 2 M CaCl_2_ was added to each virus preparation, mixed, and incubated for 15 min at room temperature before injection of 50 *μ*L into the gastrocnemius muscle. To activate CreER in a spatially restricted manner, a 5 mg/mL 4-hydroxytamoxifen (4-OHT, Sigma, H7904) solution in 25% ethanol/75% corn oil was administered via intramuscular injection. The solution was prepared by dissolving 4-OHT in ethanol and incubating at 60°C for 2 hours with shaking. Next, corn oil was added at a 3 : 1 corn oil to ethanol ratio, with continuous shaking at 65°C for 1 hour. Aliquots were stored at −80°C. 20 *μ*L was injected into the gastrocnemius of experimental mice. All mice were anaesthetized with 2% isoflurane prior to any injection or procedure. All animal studies were performed in accordance with protocols approved by the Duke University Institutional Animal Care and Use Committee.

### 2.3. Plasmid Constructs and Cloning

The pSECC vector was kindly provided by Tyler Jacks (Koch Institute at MIT) [30]. For cloning pSECC-sgp53, BbsI was used to digest pSECC and annealed sgp53 (Table S1) was cloned into the vector. sgp53 is a previously reported sgRNA sequence targeting exon 7 of *p53* in mice [31, 32]. Sanger sequencing of the vectors was used with a sequencing primer targeting the U6 promoter in pSECC to confirm successful cloning.

### 2.4. Genotyping

Mice were genotyped using tails collected from mouse pups. Tail genomic DNA was extracted with a KAPA Mouse Genotyping Kit (KAPA Biosystems, KK7352), and PCR was performed using primers listed in Supplementary Table 1. PCR products were visualized after electrophoresis in 1% agarose gels.

### 2.5. Adenovirus Constructs and Generation

The AdFC adenoviral vector was constructed from the pX333 vector backbone [33]. For cloning pX333-FC, sgFus (Table S1) and sgChop (Table S1) were cloned into px333 through two rounds of cloning. Finally, a fragment of the pX333-FC vector including the Cas9 transgene and the 2 sgRNAs was subcloned into the Ad5 adenoviral shuttle vector as described by Maddalo et al. [33] using XhoI and EcoRI sites to generate the AdFC shuttle vector for adenovirus generation. All restriction enzymes and T4 ligase were purchased from New England Biolabs. Recombinant adenoviruses were generated by Viraquest Inc. and validated *in vitro* prior to use *in vivo*.

### 2.6. In Vivo Electroporation

After mice were anesthetized, 50 *μ*g of naked DNA plasmid diluted in sterile saline was injected into the gastrocnemius using a 31-gauge insulin syringe. A final DNA concentration of 1 *μ*g/*μ*L was used, and 50 *μ*L was delivered in each injection. Electroporation was administered as previously described [32]. Briefly, a pair of needle electrodes with a 5 mm gap was inserted into the muscle to encompass the injection site, and electric pulses were delivered using a BTX Electro Square Porator ECM830 (BTX, San Diego, CA). Three 100 V pulses were administered to each injection site. The duration of each pulse was 200 ms, and the three pulses were given 50 ms apart.

### 2.7. Immunohistochemistry

Tumors were fixed in formalin overnight, transferred to 70% ethanol, paraffin-embedded, and sectioned to 5 *μ*m thickness. Deparaffinized and rehydrated slides were blocked with 3% hydrogen peroxide. Antigen retrieval was performed by boiling in 2% citrate-based antigen unmasking solution (VECTOR H-3300) for 15 min. Slides were blocked with 5% normal serum in PBS + 0.25% Tween-20 and incubated with primary antibodies: 1 : 500 mouse anti-CHOP (Cell Signaling #2895),1 : 50 rabbit anti-CD31 (Abcam ab28364), 1 : 100 mouse anti-desmin (Agilent M076029-2), 1 : 200 rabbit anti-vimentin (Abcam ab92547), 1 : 1 rabbit anti-S100 (Dako GA504), and 1 : 100 rabbit anti-cytokeratin (Abcam ab9377) diluted in PBS + 0.25% Tween-20 + 5% normal serum overnight at 4°C. After washing, slides were incubated with biotinylated secondary antibodies, including 1 : 200 horse anti-mouse IgG (VECTOR BA-2000) or 1 : 200 goat anti-rabbit IgG (VECTOR BA-1000) for 1 hour at room temperature. Slides were incubated with VECTASTAIN Elite ABC Reagent (VECTOR PK-7100) for 30 min at room temperature before signal was visualized with the 3,3′-diaminobenzidine (DAB) peroxidase substrate kit (VECTOR SK-4100). Tissue sections were examined by a sarcoma pathologist (D.M. Cardona).

### 2.8. Western Blot

Samples were lysed in RIPA buffer for 30 min on ice (Sigma-Aldrich, R0278), sonicated briefly, and then centrifuged at 10,000x·g for 20 min at 4°C. Protein concentration was determined for the lysate supernatant by BCA assay (Pierce, 23225). Samples were boiled in 4x Laemmli sample buffer (Bio-Rad, 1610747) at 95°C for 5 min and then cooled to room temperature before loading in a 4–20% Tris-glycine polyacrylamide gel. Samples were electrophoresed at 200 V for 30 min before transfer to nitrocellulose. Membranes were blocked in 5% nonfat dry milk in Tris-buffered saline (TBS, Corning, 46-012-CM). Next, the membranes were incubated overnight at 4°C with primary antibodies diluted in TBS-T (0.1% Tween-20): FUS, 1 : 1,000 (Abcam, ab84078); CHOP, 1 : 1,000 (Cell Signaling Technology, 2895S); actin, 1 : 10,000 (BD Biosciences, 612656); *p53*, 1 : 1,000 (Cell Signaling Technology, 32532S); and p21, 1 : 200 (Santa Cruz Biotechnology, sc-6246). The membranes were washed three times in TBS-T for 5 min before secondary antibody incubation with goat anti-rabbit IRDye800 (Li-Cor Biosciences, P/N 925-32211) and goat anti-mouse IRDye680 (Li-Cor Biosciences, P/N 925-68070) both at 1 : 10,000 dilutions in TBS-T for 1 h at room temperature. The membranes were washed three times in TBS-T for 5 min and imaged using an Odyssey CLx (Li-Cor Biosciences). Image analysis for normalization and quantification was performed using Image Studio (version 5.2, Li-Cor Biosciences, P/N 9140-500).

### 2.9. Quantitative RT-PCR

Cells were lysed with TRIzol reagent (Thermo Fisher, 15596026). RNA was isolated from samples using a Direct-zol RNA MiniPrep kit (Zymo Research, R2051). RNA samples were reverse transcribed to cDNA using an iScript Advanced cDNA Synthesis Kit (Bio-Rad, 1725038). TaqMan probes from Thermo Fisher were used for PCR: Gapdh (MM99999915), Trp53 (Mm01731290), Cdkn1a (Mm00432448), Bbc3 (Mm0051926), Mdm2 (Mm01233136), and Bax (Mm00432051). Plates were run on a QuantStudio 6 Flex Real-Time PCR System (Thermo Fisher), and data were analyzed using the comparative C_T_ method to generate expression fold-change values.

### 2.10. Radiation Treatments

Cells were cultured at least 24 hours prior to irradiation experiments. The X-RAD 160 (Precision X-ray) cell irradiation system was used at 160 kVp × 18 mA energy. An F1 filter (2 mm aluminum) was used for beam conditioning. Sample distance was set to 40 cm. For a dose of 10 Gy, calculations based on annual dosimetry determined a treatment duration of 230 seconds.

### 2.11. Tissue Culture and Cell Line Generation

NIH-3T3 cell lines were purchased from ATCC (CRL-1658) and cultured in DMEM (Thermo Fisher Scientific, 11965092) supplemented with 10% fetal bovine serum (Thermo Fisher Scientific, 16000044) and 1% antibiotic-antimycotic (Thermo Fisher Scientific, 10091148) and incubated at 37°C with 5% CO_2_ in a humidified cell culture incubator. Tumor cells were isolated from primary mouse sarcomas and expanded *in vitro* using standard tissue culture methods. Briefly, tumor tissue was minced in the cell culture hood and digested by dissociation buffer in PBS (Thermo Fisher Scientific, 14040133) containing collagenase type IV (5 mg/ml, Thermo Fisher Scientific, 17104-019), dispase (1.3 mg/ml, Thermo Fisher Scientific, 17105-041) as well as trypsin (0.05%, Thermo Fisher Scientific, 25200056) for about 1 h at 37°C. Cells were washed with PBS (Thermo Fisher Scientific, 10010023) and filtered using a 40 mm sieve (Corning, 431750) and cultured for at least four passages to deplete stroma before being used for experiments.

### 2.12. Gene Editing and Validation

For *in vitro* editing, cells were transfected with plasmid DNA using TransIT-LT1 transfection reagent (Mirus Bio, MIR2300) as per the manufacturer's instructions. Cells were harvested 48–72 hours later, and genomic DNA was extracted with a DNeasy Blood & Tissue Kit (Qiagen, 69504). For *in vivo* editing, mice were injected with AdFC virus in the gastrocnemius muscle or inguinal fat pads. 72 hours later, the muscle was isolated and genomic DNA was extracted with a DNeasy Blood & Tissue Kit. Editing was evaluated using the Surveyor Mutation Detection assay (IDT, 706020) and PCR.

### 2.13. Soft Agar Transformation Assay

1.8% Bactoagar was made with diH_2_O and autoclaved. 2x and 1x DMEM were prepared using DMEM powder (Thermo Fisher Scientific, 12100046), fetal bovine serum (Thermo Fisher Scientific, 16000044), and antibiotic-antimycotic (Thermo Fisher Scientific, 10091148). 0.6% agar was made by diluting 1.8% agar with DMEM and kept in a 42°C water bath. 3 mL of 0.6% agar was poured per well of 6-well plates and allowed to solidify in a hood for 10 minutes before transferring to an incubator. Cells were trypsinized and counted using trypan blue solution (Thermo Fisher Scientific, 15250061). 250 *μ*L of cells and 500 *μ*L of 0.6% agar were mixed and gently pipetted onto the bottom agar in each well to create the 0.4% agar top layer. 5000 cells were plated per well in triplicate for each cell line. After plating, plates were placed in an incubator and allowed to grow for 3-4 weeks. Medium was supplemented each week by adding 0.5 mL of DMEM per well to prevent drying.

### 2.14. Detection and Analysis of Chromosomal Rearrangements

PCR and ddPCR amplification of mouse Fus-Chop was performed using primers spanning the translocation junction (Supplementary Table 1). AccuPrime Taq DNA Polymerase, High Fidelity (Thermo Fisher Scientific, 12346086), was used as per the manufacturer's instructions for PCR. QX200 ddPCR EvaGreen Supermix (Bio-Rad, 1864034) was used for ddPCR as per the manufacturer's instructions. PCR products were either used directly for gel electrophoresis or cloned into the pCR4-TOPO TA vector (Thermo Fisher Scientific, K457501) for Sanger sequencing. ddPCR samples were analyzed on a QX200™ AutoDG™ Droplet Digital™ PCR System.

## 3. Results

### 3.1. Design and Generation of Conditional FUS-CHOP Mice

**Figure 1:**
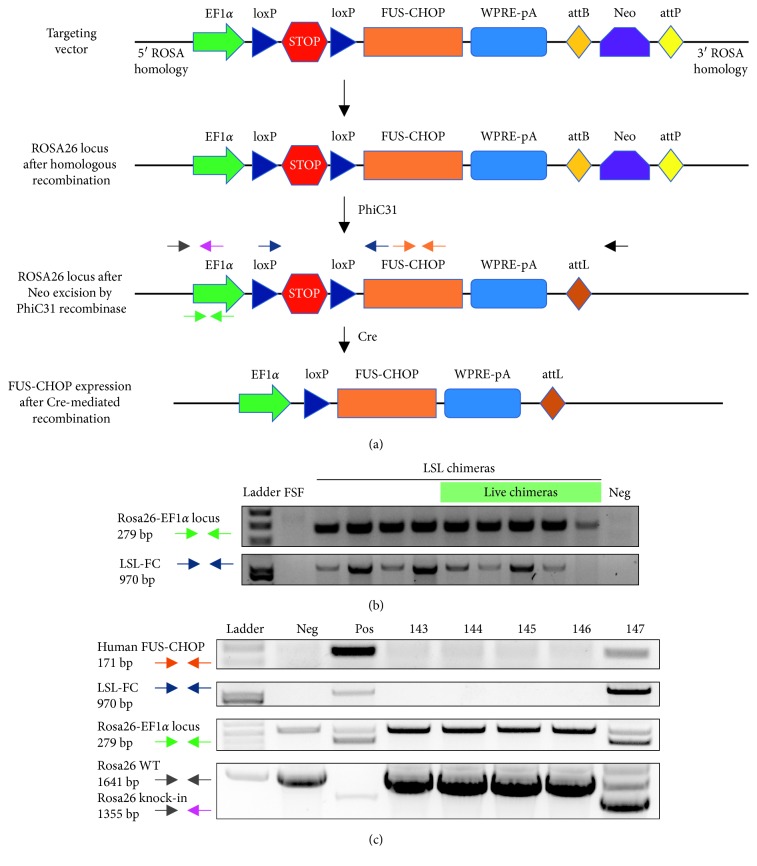
Vector targeting strategies for generation of *Rosa26* LSL-FUS-CHOP/+ mice. (a) The LSL-FUS-CHOP vector was designed to target the *Rosa26* locus. The human 7-2 FUS-CHOP cDNA is under the control of an EF1*α* promoter and a floxed STOP (LSL) cassette that prevents expression in the absence of Cre-mediated recombination. A woodchuck hepatitis virus (WHV) posttranscriptional regulatory element with a polyadenylation signal (WPRE-pA) was placed downstream of the *FUS-CHOP* cDNA to enhance transgene expression. A *Neo* selection cassette flanked by *attB* and *attP* sites was included in the targeting vector for ES cell selection. The *Neo* cassette can be deleted by PhiC31-mediated recombination of the *attB* and *attP* sites. Cre recombinase excises the STOP cassette and activates *FUS-CHOP* expression. (b) Genotyping of chimeric mice generated via ES cell injection. (c) Genotyping of pups to determine germline transmission in chimeras. Mouse 147 is a founder mouse that has the LSL-FUS-CHOP targeting vector in its germline.

To genetically engineer a mouse model with conditional expression of *FUS-CHOP*, we employed standard transgenic mouse methods starting with *FUS-CHOP* cDNA (Figure 1(a)) [6]. We used the 7-2 translocation variant of human *FUS-CHOP*, which has been reported to form tumors in mice when expressed in all tissues under an *EF-1α* promoter that is also used in this mouse model [18]. The 7-2 translocation variant, which was the first reported variant of the translocation to be discovered in MLPS, joins the first 7 exons of *FUS* in frame with exon 2 of *CHOP* via a short translated linker region originally part of the 5′UTR of *CHOP*. The *FUS-CHOP* cDNA was inserted into a vector targeting the mouse *Rosa26* locus downstream of an LSL cassette—a transcriptional STOP cassette flanked by two loxP sites (floxed). The *Rosa26* locus is widely expressed across cell types and tissues in mice. The LSL cassette enables temporally and spatially restricted activation of *FUS-CHOP* expression by Cre recombinase. A woodchuck hepatitis virus (WHV) posttranscriptional regulatory element with a polyadenylation signal (WPRE-pA) was placed downstream of the *FUS-CHOP* cDNA to enhance transgene expression. Finally, we included a Neo cassette flanked by *attB* and *attP* sites at the 3′ end of the targeting vector for positive selection of embryonic stem (ES) cell clones with neomycin.

After positive selection of ES cell clones, they were injected into blastocysts, which were subsequently transplanted into foster mothers. The chimeric pups that were born were genotyped to confirm the presence of *FUS-CHOP* cDNA in somatic genomic DNA (Figure 1(b)). Positive chimeras were crossed with wild-type 129/SvJ mice and genotyped to confirm germline transmission of the targeting vector (Figure 1(c)). Mice with confirmed germline transmission of the LSL-FUS-CHOP/+ allele became founders that were crossed with *Rosa26* PhiC-Neo/+ mice to delete the Neo cassette. Finally, these mice were crossed with wild-type 129/SvJ mice to cross out the PhiC/Neo allele, which resulted in the experimental genetically engineered mouse model with the genotype *Rosa26* LSL-FUS-CHOP/+ (LSL-FC) mice (Figure 1(a)). To confirm successful knock-in of the LSL-FUS-CHOP allele into the *Rosa26* locus and document fidelity of the cDNA sequence, we primer walked and Sanger sequenced the *Rosa26* locus in these mice (Supplementary Figure 1).

To restrict *FUS-CHOP* expression to specific mesenchymal and adipocyte precursor cell populations, we crossed *Rosa26* LSL-FUS-CHOP/+ mice with various mouse lines expressing Cre recombinase (Table 1). These mice either expressed Cre recombinase in a tissue-specific manner, or ubiquitously starting in early embryogenesis. The selection of these Cre driver candidates was based on the expression of Cre at different stages of development and adipogenesis [34–36]. *Meox2-Cre* mice express Cre in the entire animal early in embryogenesis [25, 37]. *PdgfRα-Cre* mice express the recombinase at a later stage in early mesoderm development. *Prrx1* expression occurs later than *PdgfRα* expression during mesoderm development, but before commitment to the adipocyte lineage. Importantly, the *Prrx1-Cre* mice restrict Cre activity mostly to white adipose tissue in both male and female mice [35]. Finally, *aP2* (or *Fabp4*) is highly expressed in mature adipocytes, and undifferentiated cells have low levels of *aP2* expression.

### 3.2. FUS-CHOP Expression in the *Prrx1* Lineage Drives Sarcomagenesis

**Figure 2:**
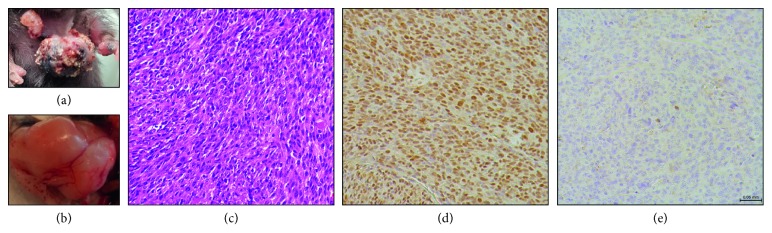
Tumor generation in the adipocytic precursor lineage and immunohistochemical characterization of *Prrx1*-Cre; *Rosa26* FUS-CHOP/+ tumors. (a) Gross image of keratoacanthomas that developed on the skin of the torso, hind limbs, and tails of mice. (b) Gross hind limb sarcomas were observed in mice after spontaneous resolution of keratoacanthomas. (c) Hematoxylin and eosin-stained section of soft tissue sarcoma. (d) FUS-CHOP IHC with CHOP antibody. (e) Section of primary mouse sarcoma from LSL-*Kras* ^G12D^; *p53^fl/fl^* mice stained with CHOP antibody.

Primary sarcomas developed in *Rosa26* LSL-FUS-CHOP/+ mice when the mice expressed Cre in specific mesenchymal lineages during development (Figure 2 and Table 2). *Rosa26* LSL-FUS-CHOP/+ mice crossed with *Meox2-Cre and PdgfRα-Cre* mice were embryonic lethal; *Rosa26* LSL-FUS-CHOP/+ mice crossed with *aP2-CreER* mice did not form any tumors up to one year after delivery of either intramuscular 4-hydroxytamoxifen (IM 4-OHT) or intraperitoneal tamoxifen (IP Tam) to activate Cre in mature adipocytes (Table 2). However, tumors formed in *Prrx1-Cre*; *Rosa26* FUS-CHOP/+ mice. *Prrx1-Cre*; *Rosa26* FUS-CHOP/+ mice developed keratoacanthomas shortly after birth (Figure 2(a), Supplementary Figure 2). Some of these masses resolved on their own, and 36% of the mice developed sarcomas in the gastrocnemius or bone (Figure 2(b)). Histologically, these tumors were high-grade undifferentiated sarcomas with spindle cells (Figure 2(c)). Immunohistochemistry shows that these tumors express FUS-CHOP in nuclei (Figure 2(d)). In contrast, tumors from a mouse model of undifferentiated pleomorphic sarcoma in *LSL-Kras* ^*G12D*^; *p53^fl/fl^* mice do not express FUS-CHOP (Figure 2(e)) [38]. Therefore, *Prrx1*-expressing cells are permissive for initiation of sarcomagenesis by FUS-CHOP.

Due to the formation of keratoacanthomas and to spatially restrict generation of tumors, we crossed *Rosa26* LSL-FUS-CHOP/+ mice with *Prrx1-CreER-GFP* mice to allow site-specific tumor generation via activation of CreER by intramuscular 4-OHT. These mice formed tumors with similar penetrance (33.3%) compared to mice with Cre expressed from the *Prrx1* promoter from birth (Table 2). However, the tumors we observed were not restricted to injection sites, possibly due to the leakiness of the CreER system. Further evidence of leakiness was the formation of tumors in noninjected *Prrx1-CreER-GFP*, *Rosa26* LSL-FUS-CHOP/+ mice.

Intramuscular injection of an adenovirus expressing Cre recombinase (AdCre) into mice with conditional activation of oncogenic *Kras* ^*G12D*^ and deletion of *p53* (*LSL-Kras* ^*G12D*^; *p53^fl/fl^*) generates primary soft tissue sarcomas with high penetrance as we previously reported [38]. Injection of AdCre into the gastrocnemius muscle of *Rosa26* LSL-FUS-CHOP/+ mice to activate *FUS-CHOP* expression was not sufficient to form tumors (Table 2). Human MLPS tumors with the endogenous translocation have a 1 : 1 : 1 stoichiometric ratio of wild-type FUS, wild-type CHOP, and FUS-CHOP. Thus, we bred *Rosa26* LSL-FUS-CHOP/+ mice to homozygosity to more accurately mimic this stoichiometry in mice. Activation of two copies of *FUS-CHOP* via AdCre delivery in *Rosa26* LSL-FUS-CHOP/LSL-FUS-CHOP mice was also insufficient to generate tumors. Interestingly, activation of FUS-CHOP with simultaneous deletion of *p53* via AdCre delivery in *Rosa26* LSL-FUS-CHOP/+, *p53^fl/fl^* mice was sufficient to generate tumors (Table 2). Similarly, when two copies of FUS-CHOP were activated with *p53* co-deletion, tumors also formed. Most importantly, because tumors did not form in *Rosa26* LSL-FUS-CHOP/+ or *Rosa26* LSL-FUS-CHOP/LSL-FUS-CHOP mice in the presence of intact *p53*, these data suggest that FUS-CHOP-driven sarcomas in mice are dependent on inactivation of *p53* or the *p53* pathway.

### 3.3. Molecular Characterization of FUS-CHOP-Driven Tumors Shows *p53* Pathway Dependency

**Figure 3:**
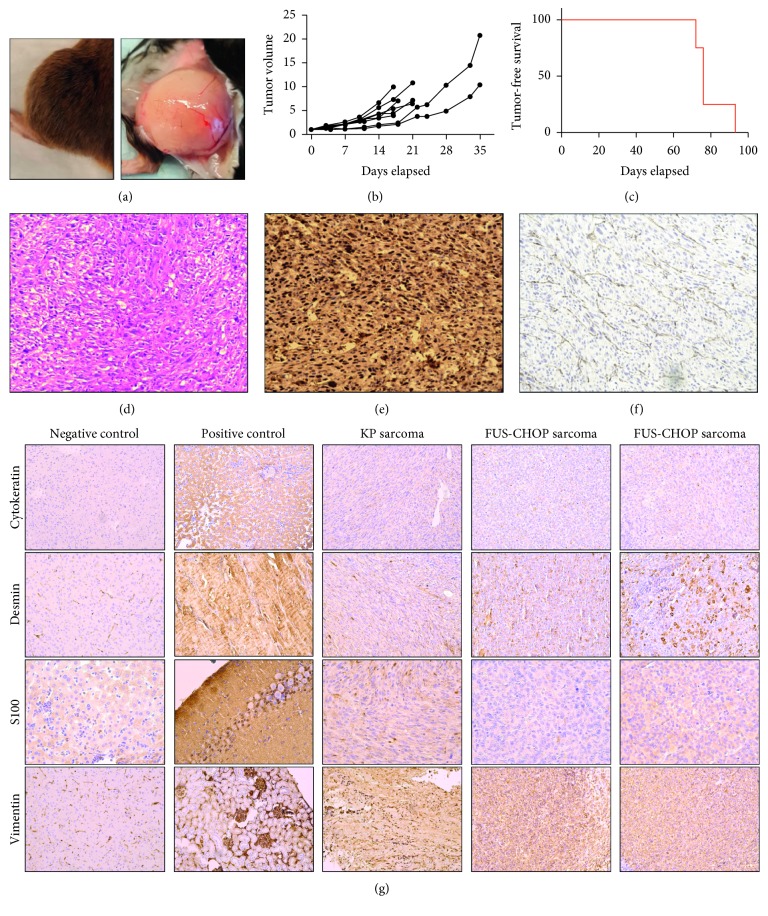
FUS-CHOP-positive primary tumors. pSECC-sgp53 IVE; *Rosa26* FUS-CHOP/+ tumors. (a) Gross images of tumors generated via pSECC-sgp53 *in vivo* electroporation. (b) Tumor growth over time. (c) Tumor-free survival. (d) H&E: IHC with (e) CHOP antibody and (f) CD31 antibody in tumors generated via pSECC-sgp53 *in vivo* electroporation. (g) Tissue lineage classification of FUS-CHOP-driven sarcomas and *Kras^G12D^*; *p53^−/−^*(KP) murine undifferentiated pleomorphic sarcomas by IHC. Negative control tissues: brain (cytokeratin/desmin/vimentin) and liver (S100). Positive control tissues: liver (cytokeratin), muscle (desmin), brain (S100), and kidney (vimentin).

To avoid keratoacanthomas and to temporally and spatially restrict tumor formation to develop a preclinical model to study FUS-CHOP-driven sarcomagenesis *in vivo*, we generated the *Rosa26* FUS-CHOP; *p53* (FCP) model, which delivers a plasmid (pSECC-sgp53) that contains Cre to activate expression of FUS-CHOP, and Cas9 and a *p53* single guide RNA (sgRNA), sgp53, to create insertions/deletions (indels) in *p53* (Figure 3) [30]. Delivering pSECC-sgp53, but not a control pSECC plasmid with a scrambled sgRNA (sgScr), into the gastrocnemius muscle via electroporation generates primary sarcomas with 100% penetrance in approximately 9–12 weeks (Figures 3(b) and 3(c), Table 2). CD31 staining of tumors revealed branching “crow's feet” vasculature in this tumor model (Figure 3(f)). The “crow's feet” vasculature is a pathognomonic feature of MLPS [2]. However, the tumors lack the myxoid stroma background found in human MLPS (Figure 3(d)). Rather, histologically, these tumors resemble high-grade sarcomas with high cellularity. To further characterize the FUS-CHOP-driven tumors, we performed IHC for markers expressed in different tissue lineages (Figure 3(g)). *Kras^G12D^*; *p53* ^−/−^(KP) murine undifferentiated pleomorphic sarcomas were negative for cytokeratin and were intermixed with scattered dendritic type cells appearing as focal positive detection of S100. In contrast, the FUS-CHOP tumors were negative for cytokeratin and S100, which shows that these tumors likely do not originate from epithelial or neural crest lineages. Furthermore, because CD31 IHC (Figure 3(f)) was only positive in the vasculature and not in tumor cells, the tumors are unlikely to be derived from endothelial cells. However, both the KP and FUS-CHOP tumors were positive for vimentin consistent with a mesenchymal origin and patchy positive for desmin in areas of the tumors.

**Figure 4:**
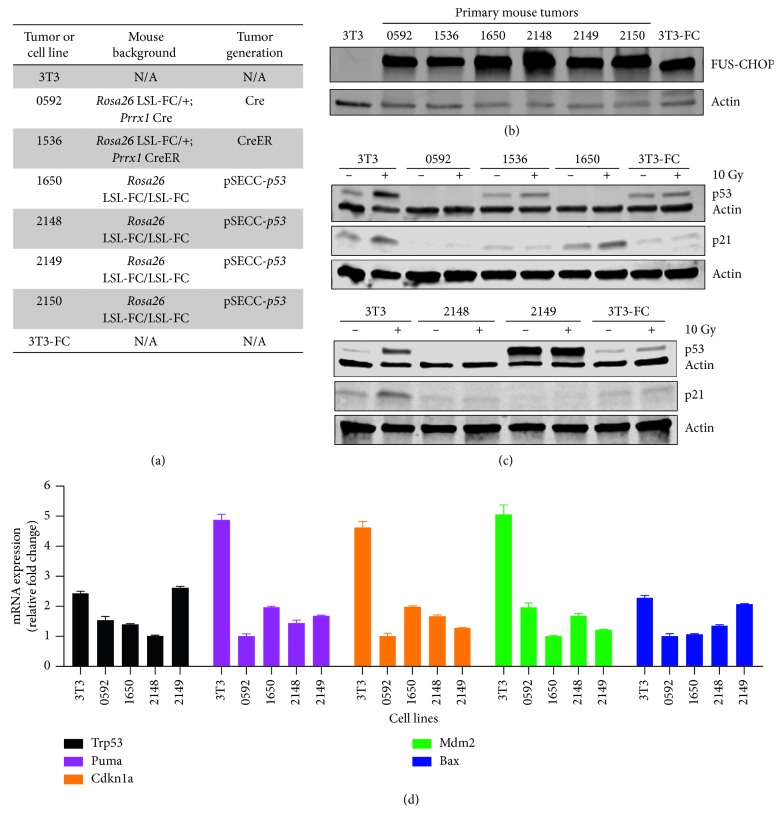
*p53* signaling and FUS-CHOP expression in FUS-CHOP-driven primary tumor cell lines. (a) Mouse background and tumor generation methods for primary tumors. (b) FUS-CHOP expression in primary mouse tumors and cell lines. (c) *p53* and *p21* expression 1 hour post-10 Gy irradiation in primary mouse tumors generated in different mouse backgrounds. 3T3 cells are mouse fusion negative controls, and 3T3-FC are mouse fusion positive controls for *p53* signaling. (d) mRNA expression of *p53* target genes 1 hour after treatment with 10 Gy ionizing radiation in NIH-3T3 and FUS-CHOP-driven tumor cell lines.

To further investigate the role of *p53* in FUS-CHOP-driven sarcomas, we interrogated *p53* signaling in cell lines derived from primary tumors with different *p53* status (Figure 4(a)). For example, tumors 0592 and 1536 represent two tumors that formed without intentional deletion of *p53*. In contrast, tumors 1650, 2148, 2149, and 2150 were formed with CRISPR-mediated indels in *p53*. These tumors all had robust expression of FUS-CHOP protein (Figure 4(b)). NIH-3T3 cells served as a negative control for FUS-CHOP expression, and a gene-edited 3T3 cell line harboring Fus-Chop at the endogenous locus served as a positive control (3T3-FC). We generated stable cell lines from these tumors and irradiated cells with 10 Gy X-ray radiation to activate *p53*. After 1 hour, we harvested the cells and performed western blots for *p53* and the *p53* transcriptional target p21 to characterize the status of *p53* and the *p53* pathway, respectively (Figure 4(c)). Western blots showed that *p53* is not expressed in tumor cell lines 0592, 1650, and 2148. While indels in *p53* were intentionally engineered *in vivo* via CRISPR/Cas9 when tumors 1650 and 2148 were initiated, tumor 0592 may have lost *p53* through clonal selection *in vivo*. The cell line derived from tumor 2149 shows robust *p53* expression even without radiation. This tumor was engineered *in vivo* with CRISPR/Cas9, and the high baseline *p53* expression suggests that a *p53* stabilizing point mutation may have been generated via nonhomologous end-joining (NHEJ) repair after CRISPR/Cas9 editing. Because p21 is not induced in this cell line after irradiation, the overexpressed *p53* appears to be nonfunctional. Although *p53* expression and induction were detected in the cell line derived from tumor 1536, *p53* in this cell line failed to induce p21 expression, which suggests that the expressed *p53* is nonfunctional. To interrogate *p53* signaling in the tumor cell lines more quantitatively, we performed qRT-PCR to measure the change in expression of several *p53* target genes such as *Puma*, *Cdkn1a (p21)*, *Mdm2*, and *Bax* 1 hour after radiation (Figure 4(d)). Induction of *p53* target gene expression was impaired in the FUS-CHOP cell lines, but not in the 3T3 positive control cell line, which has functional *p53*. We also observed high baseline p21 levels in one cell line derived from a FUS-CHOP-driven tumor (1650). This cell line did not express p53 protein, yet *p21* activation was observed after irradiation through a *p53*-independent mechanism. Taken together, these data suggest that, in FUS-CHOP-driven tumors in mice, either *p53* itself or its downstream signaling must be compromised for tumorigenesis. In the model initiated with CRISPR/Cas9 gene editing using a *p53* sgRNA, *p53* was inactivated at tumor initiation. In the other models in which Cre/loxP technology activates expression of FUS-CHOP without directly targeting *p53*, it appears that clonal selection drives *p53* pathway inactivation during tumorigenesis *in vivo*.

### 3.4. Generation of Endogenous Translocations *In Vitro*

**Figure 5:**
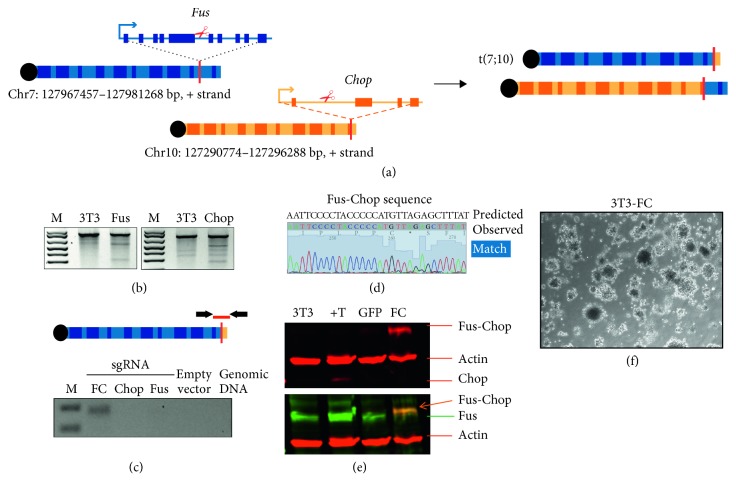
Generation of endogenous FUS-CHOP translocation with CRISPR technology. (a) Schematic of generating chromosomal rearrangements with CRISPR/Cas9 technology. *Fus* and *Chop* introns were targeted in 3T3 cells with sgRNAs for Cas9-mediated cleavage and formation of double-stranded breaks. Repair of these breaks resulted in endogenous translocations in a proportion of cells, which were single cell sorted and expanded for characterization and screening for translocation status via PCR. (b) Surveyor assay for sgFus and sgChop validation. (c) Detection of translocation by PCR shows translocation products only when both *Fus* and *Chop* sgRNAs are used. (d) Sanger sequencing of *Fus-Chop* junction PCR. (e) Western blot shows colocalization of Fus and Chop antibodies in the single cell clone (FC lane, orange arrow). (f) Soft agar assay for transformation shows robust colony formation by 3T3-FC cells.

In addition to generating genetically engineered mouse models for the conditional expression of FUS-CHOP in a tissue-specific manner, we also devised a strategy to generate endogenous Fus-Chop chromosomal rearrangements *in vivo* using CRISPR/Cas9 technology (Figure 5). This approach would enable the rapid, site-specific generation of autochthonous tumors in mice driven by the FUS-CHOP translocation expressed from the endogenous Fus locus with haploinsufficiency for FUS and CHOP. To generate the FUS-CHOP fusion, we modified the dual-guide plasmid system described by Maddalo et al. [33], hereafter referred to as pX333-FC, to deliver sgRNAs targeting *Fus* and *Chop* at intron 7 and intron 1, respectively (Figure 5(a)). To aid in selection and enrichment of cells harboring the translocation, we cloned versions of pX333-FC containing either a puromycin resistance marker or GFP to use in transfections.

NIH-3T3 cells were transfected with pX333-FC, and genomic DNA was assayed using Surveyor endonuclease to detect mutations and confirm Cas9 activity at the targeted loci (Figure 5(b)). At sites of Cas9-mediated double-stranded cleavage, indels form as a result of nonhomologous end-joining (NHEJ) repair. PCR for the amplicon of interest followed by repeated cycles of denaturing and annealing will generate amplicon heterodimers that have a mismatch. Surveyor endonuclease specifically cleaves DNA at sites of mismatch and will produce two fragments when run on a gel. Here, the presence of cleavage products demonstrates successful and specific CRISPR/Cas9 activity. Furthermore, the t(7;10) translocation was detected by PCR (Figure 5(c)) and confirmed with sequencing (Figure 5(d)). This experiment demonstrated that pX333-FC can be used to generate the Fus-Chop translocation in NIH-3T3 cells. We have successfully used this approach to generate the fusion in other cell lines derived from KP mouse sarcomas [38] (Supplementary Figure 3(a)).

Using this approach, we generated a stable Fus-Chop-expressing NIH-3T3 cell line using CRISPR/Cas9 technology. First, we transfected the pX333-FC-GFP plasmid into NIH-3T3 cells and flow sorted single GFP-positive cells into 96-well plates. Colonies that formed from single cells were screened by PCR for the fusion transcript junction. Furthermore, western blotting using two different antibodies to Fus and Chop demonstrated successful costaining at approximately 70 kDa, consistent with previously published data on the FUS-CHOP oncoprotein and predicted size (Figure 5(e)).

To assess the transformation capability of these cell lines, we performed transformation assays in soft agar. Only NIH-3T3 cells that were positive for FUS-CHOP after genome editing could form colonies in soft agar (Figure 5(f), Supplementary Figure 3(b)). These cells also formed tumors by 20 days after intramuscular injection into the hind limb of 3 of 3 NCr nude mice (Supplementary Figure 3(c)). No tumors formed following allograft of control NIH-3T3 cells lacking FUS-CHOP for the duration of the allograft experiment up to 6 weeks.

### 3.5. CRISPR-Mediated Chromosomal Rearrangements *In Vivo*

Determining the efficiency of generating endogenous chromosomal rearrangements via CRISPR/Cas9 may have important implications for establishing an *in vivo* model using this approach because targeting a critical number of progenitor cells to induce the oncogenic translocation may be a technical obstacle for initiating a tumor in mice. To assess the efficiency of generating the endogenous Fus-Chop translocation *in vitro*, we used pX333-FC-GFP to generate KP sarcoma cell lines expressing endogenous Fus-Chop. We screened 229 single KP cells to isolate 9 single cell clones that were positive for the FUS-CHOP translocation (approximately 3.9% efficiency) (Supplementary Figure 3(a)).

**Figure 6:**
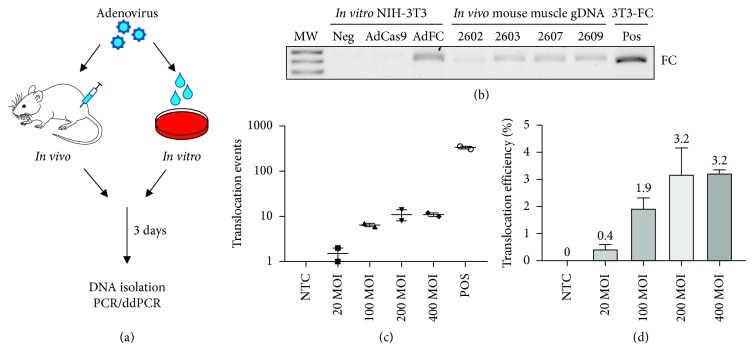
Determination of endogenous chromosomal rearrangement efficiency *in vitro* and *in vivo*. (a) Schematic of *in vitro* and *in vivo* translocation generation via adenovirus. (b) *In vitro* and *in vivo* PCR validation of AdFC-generated t(7;10). (c) ddPCR quantification of translocation events *in vitro*. (d) ddPCR quantification of translocation efficiency *in vitro*.

We attempted to generate the *Fus-Chop* translocation at the endogenous locus in wild-type mice by using an adenovirus, AdFC, to deliver two sgRNAs with Cas9 to target the respective introns of *Fus* and *Chop* (Figure 5(a)). We cloned and validated AdFC to generate autochthonous tumors expressing Fus-Chop in wild-type mice. AdFC successfully generated the endogenous *Fus-Chop* translocation *in vitro* and *in vivo*, which was detected by translocation junction-specific PCR (Figure 6), but we never observed tumors in wild-type 129/SvJ mice injected with AdFC (Table 2). Because we were able to detect the *Fus-Chop* translocation in genomic DNA from the gastrocnemius muscles of mice injected with AdFC, this suggests that the t(7;10) translocation was successfully generated in muscle cells but was not sufficient for transformation. Because our prior experiments suggested that *p53* inactivation is important for FUS-CHOP-driven tumorigenesis in mice, we attempted to initiate tumors by generating a t(7;10) in *p53^fl/fl^* mice by co-administering AdCre, to delete *p53*, and AdFC, to engineer the translocation. None of the mice coinjected with these viruses developed tumors (Table 2). These data and the results from our transgenic mouse models suggest that translocation efficiency may have been too low in the *in vivo* setting to generate tumors. To more precisely measure the efficiency of generating CRISPR-mediated translocations, we used droplet digital PCR (ddPCR). We transduced NIH-3T3 cells *in vitro* at different multiplicities of infection (MOI) of AdFC virus and harvested genomic DNA for ddPCR quantification (Supplementary Figure 4). Translocation efficiency was confirmed to be approximately 3% *in vitro* (Figures 6(c) and 6(d)). We hypothesize that the efficiency may even be lower *in vivo* and is a bottleneck for generation of some cancers that require multiple genetic mutations for tumorigenesis, such as FUS-CHOP-driven tumors.

## 4. Discussion

Specific sarcoma subtypes harbor specific gene fusions, which likely drive sarcomagenesis in these tumors [39]. Chromosomal rearrangements have long been recognized as important for diagnosis and treatment of sarcomas, but the biological role of these gene fusions in sarcomagenesis is less well understood. Here, we generated and characterized multiple genetically engineered mouse models of FUS-CHOP-driven sarcoma to dissect the molecular events required for sarcomagenesis *in vivo*.

We successfully generated sarcomas in mice that are driven by FUS-CHOP, recapitulate the “crow's feet” vasculature of human MLPS histologically, and resemble high-grade soft tissue sarcomas. These FUS-CHOP-driven tumors are primarily mesenchymal in origin and do not express markers of differentiation for epithelial, vascular, or neural crest tissues based on IHC (Figures 3(f)–3(g)). In mice, we find that alterations in the *p53* pathway are required for FUS-CHOP-mediated tumorigenesis. Using *in vivo* electroporation with CRISPR/Cas9 technology, we also generated a spatially and temporally restricted FUS-CHOP-driven sarcoma model that forms with high penetrance. Because different sarcomas subtypes appear to arise from different mesenchymal cell lineages, we hypothesized that FUS-CHOP-driven sarcomagenesis may be tissue-specific.

To approach this question, we used a conditional gene expression strategy that allowed us to restrict FUS-CHOP oncogene expression to specific tissue lineages *in vivo* (Figure 1 and Table 1). Our mouse models showed that specific tissues and developmental stages are permissive for FUS-CHOP-driven tumor formation. Specifically, tissues expressing *Prrx1*, a marker of early mesoderm and mesenchymal progenitor cells in several tissues [27], were permissive for FUS-CHOP-driven tumors. We discovered that early embryonic expression of FUS-CHOP in *Meox2-Cre* and *PdgfRα-Cre* mice was lethal. Furthermore, expression of FUS-CHOP in *aP2*-expressing cells, such as mature adipocytes, did not form tumors. Importantly, although our mouse models identify *Prrx1*-expressing cells as a source of FUS-CHOP-driven tumors, cells expressing *PdgfRα* cannot be ruled out as a potential tumor-initiating cell for FUS-CHOP-driven tumors. In *PdgfRα-Cre* mice, all cells expressing *PdgfRα* in the early embryo express FUS-CHOP resulting in embryonic lethality, but adult mouse tissues that express *PdgfRα* may still be permissive for transformation by FUS-CHOP. Despite this caveat, we demonstrate that not all tissues are permissive for transformation by FUS-CHOP through experiments using three mouse models (Table 2). First, the *aP2-CreER*, *Rosa26* FUS-CHOP/+ mouse demonstrates that, in differentiated adipose tissue expressing *aP2*, FUS-CHOP is not sufficient to initiate tumorigenesis. Second, we show that, in *Rosa26* LSL-FUS-CHOP/+ mice, activation of FUS-CHOP in gastrocnemius muscle via AdCre administration is also not sufficient for transformation. In human MLPS tumors, in addition to the formation of the FUS-CHOP translocation, one wild-type copy of both FUS and CHOP is lost and the stoichiometric ratio of FUS to CHOP to FUS-CHOP is 1 : 1 : 1. Thus, we used a third model of FUS-CHOP-driven sarcoma to ask if gene dosage was important for FUS-CHOP-driven transformation. Activation of two copies of FUS-CHOP in the gastrocnemius muscle of *Rosa26* LSL-FUS-CHOP/LSL-FUS-CHOP mice via AdCre administration did not form tumors. Taken together, these models demonstrate that FUS-CHOP interferes with normal development when expressed early in embryogenesis and that FUS-CHOP expression alone in differentiated cells is not sufficient to initiate sarcomagenesis. Additionally, gene dosage cannot overcome genetic or epigenetic barriers that restrict the tissue-specific nature of FUS-CHOP-driven sarcomagenesis.

We conclude from our *in vivo* experiments that FUS-CHOP is not sufficient to drive tumorigenesis in all cell types and that the *Prrx1* lineage is permissive for transformation. Next, we hypothesized that, in addition to tissue specificity, there may be other molecular determinants of FUS-CHOP-driven sarcomagenesis. *p53* has been reported to be mutated in MLPS and is the most frequently mutated gene across all sarcomas [23, 40–42]. Overexpression of *p53* correlates with reduced metastatic disease-free survival in localized MLPS [4], and models of MLPS have been generated on *p53* null backgrounds [23, 43]. These studies suggest that *p53* plays an important role in MLPS initiation and development. However, the earliest mouse models of MLPS did not require *p53* deletion or inactivation, suggesting that inactivation of *p53* is not required for sarcomagenesis [9, 44]. To clarify the role of *p53* in FUS-CHOP-driven sarcomagenesis, we crossed *Rosa26* LSL-FUS-CHOP/+ and *Rosa26* LSL-FUS-CHOP/LSL-FUS-CHOP mice with *p53^fl/fl^* mice (Table 2). Administration of AdCre successfully generated tumors only in mice that had floxed *p53* alleles and *p53* deleted in tumors. Moreover, despite successfully modeling FUS-CHOP-driven sarcomas in the *Prrx1* tissue lineage without targeting *p53* for mutation, western blot analysis of primary tumor cell lines derived from these tumors revealed that *p53* protein in these tumors was either absent or nonfunctional (Figure 4(c)). Induction of gene expression in several *p53* downstream target genes after ionizing radiation was also absent in FUS-CHOP-driven tumor cell lines (Figure 4(d)). Thus, these data suggest that FUS-CHOP-driven sarcomagenesis in mice is *p53* pathway-dependent and requires inactivation of *p53*. While it is well established that *p53* is important for preventing tumorigenesis, conditional site-specific deletion or knockout of *p53* is not sufficient for sarcomagenesis in mice [32, 38]. In contrast, the FUS-CHOP-driven sarcoma model achieves 100% penetrance in the *Rosa26* LSL-FUS-CHOP/LSL-FUS-CHOP; *p53^fl/fl^* mice in 2-3 months. This difference indicates that FUS-CHOP expression contributes to the development of sarcomas in mice.

Genetically engineered mouse models expressing human transgenes are powerful tools for dissecting mechanisms of tumorigenesis. However, the regulation of an exogenous transgene may differ from genes expressed at the endogenous locus. To most accurately model FUS-CHOP-driven sarcomagenesis, we hypothesized that CRISPR/Cas9 technology could be applied to generate the t(7;10) translocation (equivalent to t(12;16) in humans) at the endogenous locus in mice. Advantages of this approach include expression of the gene fusion from its endogenous promoter at physiological levels under wild-type *Fus* promoter/enhancer regulation, and simultaneous editing of the genome to be haploinsufficient for wild-type *Fus* and wild-type *Chop*. We generated and validated an adenovirus, AdFC, to deliver Cas9 and sgRNAs for *Fus* and *Chop* to generate t(7;10) (Figure 6). While this approach was effective *in vitro*, no tumors formed *in vivo* (Figure 5, Table 2). Based on our prior findings, we also hypothesized that the lack of simultaneous *p53* inactivation may have prevented tumor formation. To test this hypothesis, we co-delivered AdCre and AdFC into *p53^fl/fl^* mice, but we observed no tumors (Table 2). Therefore, we reasoned that low *in vivo* translocation efficiency may be a technical barrier for CRISPR-mediated tumorigenesis in this system. While CRISPR/Cas9 knockout mouse models require only one hit per gene, for translocation models, each cell that is edited only has two opportunities to form rearrangements before the PAM sites are destroyed and no further edits are possible. Additionally, NHEJ repair must produce a product that is in frame without a large deletion, which further decreases the efficiency of generating a functional fusion protein. ddPCR quantification of translocation events and *in vivo* detection of translocation suggest that although translocations are forming in mice, not enough cells that are permissive for FUS-CHOP-driven tumorigenesis are edited to form a tumor. Alternatively, though rearrangements are occurring in cells, these translocations are not occurring in a permissive cell type. Additional studies into the role of DNA topology and species-specific differences between humans and mice may further illuminate the biology of translocation-mediated sarcomagenesis.

In summary, we report the generation of multiple conditional mouse models of FUS-CHOP-driven sarcoma and show that *Prrx1*-expressing cells are a potential source of myxoid liposarcoma. We also show that differentiated fat cells are not permissive for transformation by FUS-CHOP and that *p53* pathway inactivation is required for sarcomagenesis in mice. We demonstrate that CRISPR/Cas9 can be used to rapidly generate the FUS-CHOP translocation *in vivo* and *in vitro*, but translocation efficiency, developmental stage, and tissue specificity can be barriers to tumorigenesis. These genetically engineered mouse models and novel CRISPR tools will accelerate the study of FUS-CHOP translocation-driven sarcomagenesis and can serve as valuable preclinical models of FUS-CHOP-driven sarcomas to study the response to radiation therapy, chemotherapy, and novel therapeutics.

## Figures and Tables

**Table 1:** Cre and CreER drivers of adipocytic lineage.

Mouse line	Cre or CreER driver	Purpose
*Meox2-Cre*	Embryonic/epiblast	Whole animal expression
*PdgfRα-Cre*	Embryonic/mesoderm	Early mesoderm-specific expression
*Prrx1-Cre*	Mesoderm progenitor	Mesoderm-specific expression
*Prrx1-CreER-GFP*	Mesoderm progenitor	Mesoderm-specific expression
*aP2-CreER*	Mature adipocyte	Differentiated fat-specific expression

**Table 2:** Mouse modeling approaches and tumor penetrance.

	CRISPR/Cre activation	Tumors	Total mice	Penetrance
*Tissue-specific Cre models*				
*Meox2-Cre*, *Rosa26* LSL-FUS-CHOP/+	Endogenous Cre	Embryonic lethal		
*PdgfRα-Cre*, *Rosa26* LSL-FUS-CHOP/+	Endogenous Cre	Embryonic lethal		
*Prrx1-Cre*, *Rosa26* LSL-FUS-CHOP/+	Endogenous Cre	4	11	36.4%
*Prrx1-CreER-GFP*, *Rosa26* LSL-FUS-CHOP/+	CreER, IM 4-OHT	2	6	33.3%
*aP2-CreER*, *Rosa26* LSL-FUS-CHOP/+	CreER, IP tamoxifen	0	9	0.0%

*Site-specific Cre models*				
*Rosa26* LSL-FUS-CHOP/+	Adenovirus	0	13	0.0%
*Rosa26* LSL-FUS-CHOP/+, *p53^fl/fl^*	Adenovirus	2	11	18.2%
*Rosa26* LSL-FUS-CHOP/LSL-FUS-CHOP	Adenovirus	0	29	0.0%
*Rosa26* LSL-FUS-CHOP/LSL-FUS-CHOP, *p53^fl/fl^*	Adenovirus	7	29	24.1%
*Rosa26* LSL-FUS-CHOP/LSL-FUS-CHOP, pSECC-sgp53	CRISPR, electroporation	21	21	100.0%
*Rosa26* LSL-FUS-CHOP/LSL-FUS-CHOP, pSECC-sgScr	CRISPR, electroporation	0	3	0.0%

*In vivo CRISPR model*				
129/SvJ, AdFC-Cas9	CRISPR, Adenovirus	0	31	0.0%
*p53^fl/fl^*, AdCre, AdFC-Cas9	CRISPR/Cre, Adenovirus	0	4	0.0%

*UPS model*				
*LSL-Kras ^G12D/+^*, *p53^fl/fl^*	Adenovirus	10	10	100.0%

## Data Availability

The data used to support the findings of this study are included within the article and supplementary information file(s).

## Supplementary Materials

Supplementary Materials Supplementary Table 1. Oligo sequences for all primers and sgRNAs used. Supplementary Figures 1–4. Sanger sequencing, histology, clone screening, transformation assays, and ddPCR raw counts.

## References

[1] Chung P. W. M., Deheshi B. M., Ferguson P. C. (2009). Radiosensitivity translates into excellent local control in extremity myxoid liposarcoma. *Cancer*.

[2] de Vreeze R. S. A., de Jong D., Haas R. L., Stewart F., van Coevorden F. (2008). Effectiveness of radiotherapy in myxoid sarcomas is associated with a dense vascular pattern. *International Journal of Radiation Oncology ∗ Biology ∗ Physics*.

[3] Wylie J. (2004). Pathology and genetics of tumours of soft tissue and bone. Published 2002, 1st edition, ISBN 92 832 24132. *Surgical Oncology*.

[4] Antonescu C. R., Tschernyavsky S. J., Decuseara R. (2001). Prognostic impact of *P53* status, TLS-CHOP fusion transcript structure, and histological grade in myxoid liposarcoma: a molecular and clinicopathologic study of 82 cases. *Clinical Cancer Research*.

[5] Panagopoulos I., Mandahl N., Ron D. (1994). Characterization of the CHOP breakpoints and fusion transcripts in myxoid liposarcomas with the 12;16 translocation. *Cancer Research*.

[6] Crozat A., Åman P., Mandahl N., Ron D. (1993). Fusion of CHOP to a novel RNA-binding protein in human myxoid liposarcoma. *Nature*.

[7] Killela P. J., Reitman Z. J., Jiao Y. (2013). TERT promoter mutations occur frequently in gliomas and a subset of tumors derived from cells with low rates of self-renewal. *Proceedings of the National Academy of Sciences*.

[8] Joseph C. G., Hwang H., Jiao Y. (2014). Exomic analysis of myxoid liposarcomas, synovial sarcomas, and osteosarcomas. *Genes, Chromosomes and Cancer*.

[9] Pérez-Mancera P. A., Pérez-Losada J., Sánchez-Martín M. (2002). Expression of the FUS domain restores liposarcoma development in CHOP transgenic mice. *Oncogene*.

[10] Jauhiainen A., Thomsen C., Strömbom L. (2012). Distinct cytoplasmic and nuclear functions of the stress induced protein DDIT3/CHOP/GADD153. *PLoS One*.

[11] Marciniak S. J., Yun C. Y., Oyadomari S. (2004). CHOP induces death by promoting protein synthesis and oxidation in the stressed endoplasmic reticulum. *Genes & Development*.

[12] Zinszner H., Kuroda M., Wang X. (1998). CHOP is implicated in programmed cell death in response to impaired function of the endoplasmic reticulum. *Genes & Development*.

[13] Papathanasiou M. A., Kerr N. C., Robbins J. H. (1991). Induction by ionizing radiation of the gadd45 gene in cultured human cells: lack of mediation by protein kinase C. *Molecular and Cellular Biology*.

[14] Hicks G. G., Singh N., Nashabi A. (2000). Fus deficiency in mice results in defective B-lymphocyte development and activation, high levels of chromosomal instability and perinatal death. *Nature Genetics*.

[15] Kuroda M., Sok J., Webb L. (2000). Male sterility and enhanced radiation sensitivity in TLS-/- mice. *The EMBO Journal*.

[16] Boulay G., Sandoval G. J., Riggi N. (2017). Cancer-specific retargeting of BAF complexes by a prion-like domain. *Cell*.

[17] Schwartz J. C., Cech T. R., Parker R. R. (2015). Biochemical properties and biological functions of FET proteins. *Annual Review of Biochemistry*.

[18] Pérez-Losada J., Pintado B., Gutiérrez-Adán A. (2000). The chimeric FUS/TLS-CHOP fusion protein specifically induces liposarcomas in transgenic mice. *Oncogene*.

[19] de Graaff M. A., Yu J. S. E., Beird H. C. (2016). Establishment and characterization of a new human myxoid liposarcoma cell line (DL-221) with the FUS-DDIT3 translocation. *Laboratory Investigation*.

[20] Riggi N., Cironi L., Provero P. (2006). Expression of the FUS-CHOP fusion protein in primary mesenchymal progenitor cells gives rise to a model of myxoid liposarcoma. *Cancer Research*.

[21] Rodriguez R., Rubio R., Menendez P. (2012). Modeling sarcomagenesis using multipotent mesenchymal stem cells. *Cell Research*.

[22] Rodriguez R., Tornin J., Suarez C. (2013). Expression of FUS-CHOP fusion protein in immortalized/transformed human mesenchymal stem cells drives mixoid liposarcoma formation. *Stem Cells*.

[23] Rodriguez R., Rubio R., Gutierrez-Aranda I. (2011). FUS-CHOP fusion protein expression coupled to *p53* deficiency induces liposarcoma in mouse but not in human adipose-derived mesenchymal stem/stromal cells. *Stem Cells*.

[24] Raymond C. S., Soriano P. (2007). High-efficiency FLP and ΦC31 site-specific recombination in mammalian cells. *PLoS One*.

[25] Tallquist M. D., Soriano P. (2000). Epiblast-restricted cre expression in MORE mice: a tool to distinguish embryonic vs. extra-embryonic gene function. *Genesis*.

[26] Roesch K., Jadhav A. P., Trimarchi J. M. (2008). The transcriptome of retinal müller glial cells. *Journal of Comparative Neurology*.

[27] Logan M., Martin J. F., Nagy A., Lobe C., Olson E. N., Tabin C. J. (2002). Expression of cre recombinase in the developing mouse limb bud driven by aPrxl enhancer. *Genesis*.

[28] Kawanami A., Matsushita T., Chan Y. Y., Murakami S. (2009). Mice expressing GFP and CreER in osteochondro progenitor cells in the periosteum. *Biochemical and Biophysical Research Communications*.

[29] Imai T., Jiang M., Chambon P., Metzger D. (2001). Impaired adipogenesis and lipolysis in the mouse upon selective ablation of the retinoid X receptor alpha mediated by a tamoxifen-inducible chimeric cre recombinase (Cre-ERT2) in adipocytes. *Proceedings of the National Academy of Sciences*.

[30] Sánchez-Rivera F. J., Papagiannakopoulos T., Romero R. (2014). Rapid modelling of cooperating genetic events in cancer through somatic genome editing. *Nature*.

[31] Platt R. J., Chen S., Zhou Y. (2014). CRISPR-Cas9 knockin mice for genome editing and cancer modeling. *Cell*.

[32] Huang J., Chen M., Whitley M. J. (2017). Generation and comparison of CRISPR-Cas9 and cre-mediated genetically engineered mouse models of sarcoma. *Nature Communications*.

[33] Maddalo D., Manchado E., Concepcion C. P. (2014). In vivo engineering of oncogenic chromosomal rearrangements with the CRISPR/Cas9 system. *Nature*.

[34] Uezumi A., Ikemoto-Uezumi M., Tsuchida K. (2014). Roles of nonmyogenic mesenchymal progenitors in pathogenesis and regeneration of skeletal muscle. *Frontiers in Physiology*.

[35] Krueger K. C., Costa M. J., Du H., Feldman B. J. (2014). Characterization of cre recombinase activity for in vivo targeting of adipocyte precursor cells. *Stem Cell Reports*.

[36] Jeffery E., Berry R., Church C. D. (2014). Characterization of cre recombinase models for the study of adipose tissue. *Adipocyte*.

[37] Candia A. F., Hu J., Crosby J. (1992). Mox-1 and Mox-2 define a novel homeobox gene subfamily and are differentially expressed during early mesodermal patterning in mouse embryos. *Development*.

[38] Kirsch D. G., Dinulescu D. M., Miller J. B. (2007). A spatially and temporally restricted mouse model of soft tissue sarcoma. *Nature Medicine*.

[39] Mitelman F., Johansson B., Mertens F. (2007). The impact of translocations and gene fusions on cancer causation. *Nature Reviews Cancer*.

[40] Oda Y., Yamamoto H., Takahira T. (2005). Frequent alteration ofp16INK4a/p14ARF and *p53* pathways in the round cell component of myxoid/round cell liposarcoma: *p53* gene alterations and reduced p14ARF expression both correlate with poor prognosis. *Journal of Pathology*.

[41] Pilotti S., Torre G. D., Lavarino C. (1997). Distinct MDM2/*p53* expression patterns in liposarcoma subgroups: implications for different pathogenetic mechanisms. *Journal of Pathology*.

[42] Abeshouse A., Adebamowo C., Adebamowo S. N. (2017). Comprehensive and integrated genomic characterization of adult soft tissue sarcomas. *Cell*.

[43] Charytonowicz E., Terry M., Coakley K. (2012). PPAR*γ* agonists enhance ET-743-induced adipogenic differentiation in a transgenic mouse model of myxoid round cell liposarcoma. *Journal of Clinical Investigation*.

[44] Pérez-Losada J., Sánchez-Martín M., Rodríguez-García M. A. (2000). Liposarcoma initiated by FUS/TLS-CHOP: the FUS/TLS domain plays a critical role in the pathogenesis of liposarcoma. *Oncogene*.

