# Supplementary material for: The Fusion Oncogene FUS-CHOP Drives Sarcomagenesis of High-Grade Spindle Cell Sarcomas in Mice
Source: Sarcoma. 2019 Jul 25;2019:1340261. doi: 10.1155/2019/1340261 (PMC6683777; doi:10.1155/2019/1340261)
Supplement: Supplementary Materials — Supplementary Table 1. Oligo sequences for all primers and sgRNAs used. Supplementary Figures 1–4. Sanger sequencing, histology, clone screening, transformation assays, and ddPCR raw counts. [file 1340261.f1.pdf]

Table S1. Oligo sequences for all experiments

Oligos	Sequence	Purpose	Size (bp)
5pR26_F1	TTCTGGGAGTTCTCTGCTGC	Rosa26 genotyping	279
5pEF1_R1	AGCCAGTACACGACATCACTTT	Rosa26 genotyping	279
EF1 loxP_F	TGAGGGCCCGTCGACATTTA	LSL-FC genotyping	970
5pFC_R1	TGACTGCTCTGCTGGGAATAG	LSL-FC genotyping	970
c_hFC_Fa	AGTGGTGGCTATGAACCCAG	human FC internal sequence genotyping	171
c_hFC_Ra	TGCAGTTGGATCAGTCTGGA	human FC internal sequence genotyping	171
R26wt_5F_1	CTAGGGGTTGGATAAGCCAGTA	Rosa26 locus WT genotyping	1641
R26wt_LSLFCHR_3	CAGAGAGCCTCGGCTAGGTA	Rosa26 locus WT genotyping	1641
R26wt_5F_1	CTAGGGGTTGGATAAGCCAGTA	Rosa26 locus knockin genotyping	1355
5pEF1_R1	AGCCAGTACACGACATCACTTT	Rosa26 locus knockin genotyping	1355
p53_surv_F	GGGTGAAGCTCAACAGGCTC	Surveyor assay, products: 273 bp, 478 bp	751
p53_surv_R	GGGTGAAGCTCAACAGGCTC	Surveyor assay, products: 273 bp, 478 bp	751
Fus_surv_F	GGAAGGAAGGTGTGGACTACAA	Surveyor assay, products: 597 bp, 322 bp	919
Fus_surv_R	ACGAGATCCTTGATCCCGAGG	Surveyor assay, products: 597 bp, 322 bp	919
Chop_surv_F	TCCTCAGTCCCGTCTCCTAA	Surveyor assay, products: 489 bp, 258 bp	747
Chop_surv_R	AGATCTGCCTCTCTGAGTGC	Surveyor assay, products: 489 bp, 258 bp	747
FC_F	ACTTCTGGTCTGTTCCCTGG	Translocation detection	412
FC_R	CGGCTGCTTGAGTTTCCTTT	Translocation detection	412
sgp53	GTGTAATAGCTCCTGCATGG	p53 knockout sgRNA	NA
sgScrambled	GTCATGTCACTTATCAAGTC	Negative control sgRNA	NA
sgFus	ATGTCCTCAGCACAGATGG	Fus target for translocation	NA
sgChop	CTATAAAGCTCTAACACCG	Chop target for translocation	NA

Supplemental Figures

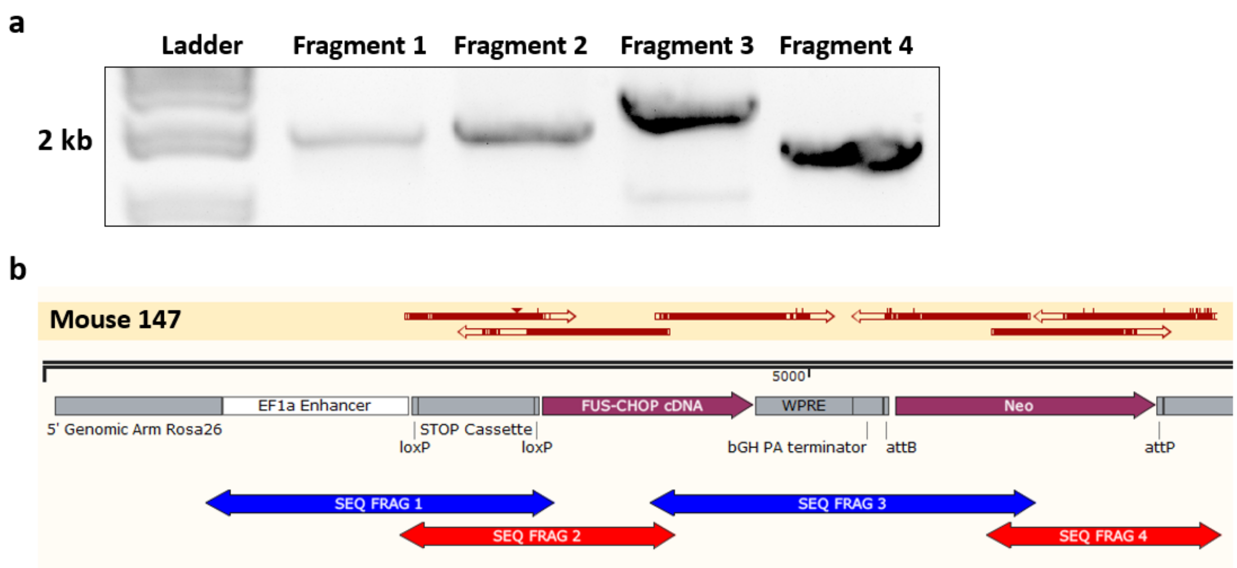

Supplemental Figure 1. Primer walking, Sanger sequencing, and alignment of the Rosa26 locus in Rosa26 LSL-FUS-CHOP mice. (a) PCR of fragments used for Sanger sequencing. (b) Alignment of sequenced fragments to the complete target vector sequence.

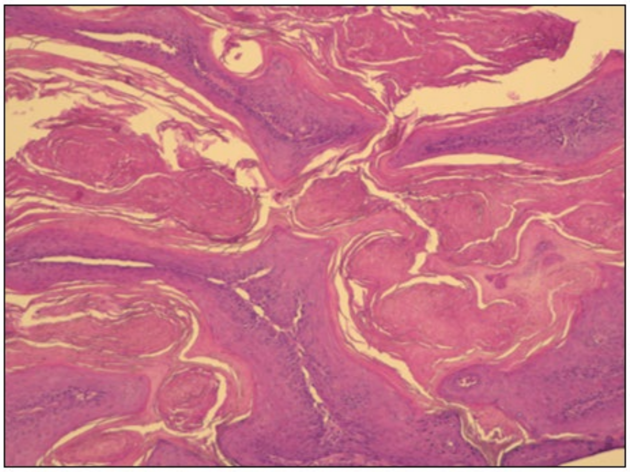

Supplemental Figure 2. Hematoxylin and eosin stained section of a mouse keratoacanthoma.

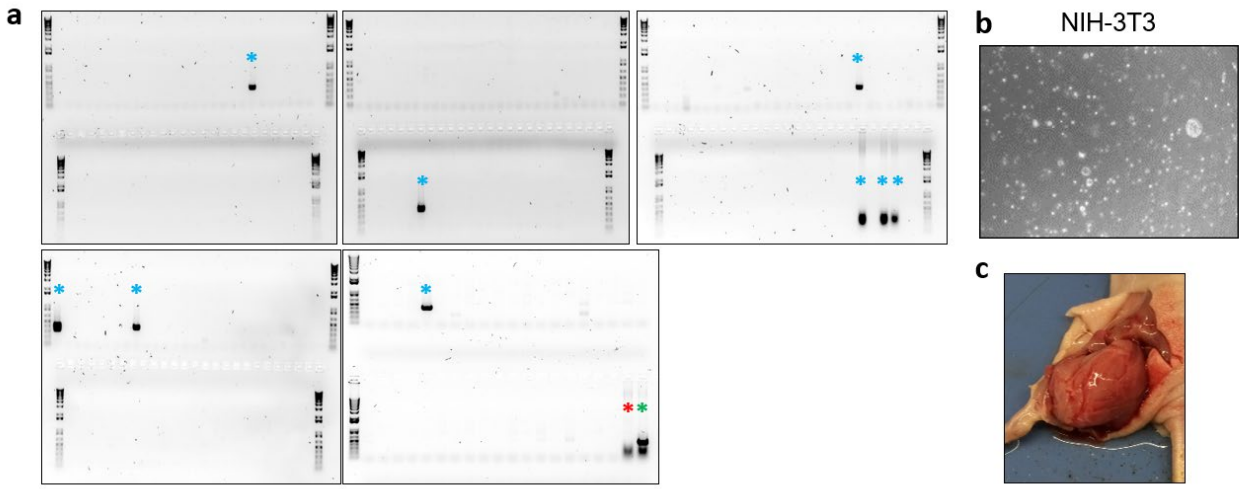

Supplemental Figure 3. FUS-CHOP CRISPR clone screening and transformation assays.

(a) Screening of KP cells transfected with pX333-FC-GFP, sorted for GFP expression, and plated to generate single cell clones. FUS-CHOP positive clones were detected via PCR for the translocation junction. 229 colonies were screened in total. Asterisks denote the negative control (red), the positive control (green), and positive clones (blue). (b) Negative control soft agar transformation assay with NIH-3T3 cells showing lack of colony formation. (c) Gross image of a tumor that developed after 3T3-FC cells were allografted into the hindlimb of NCr nude mice.

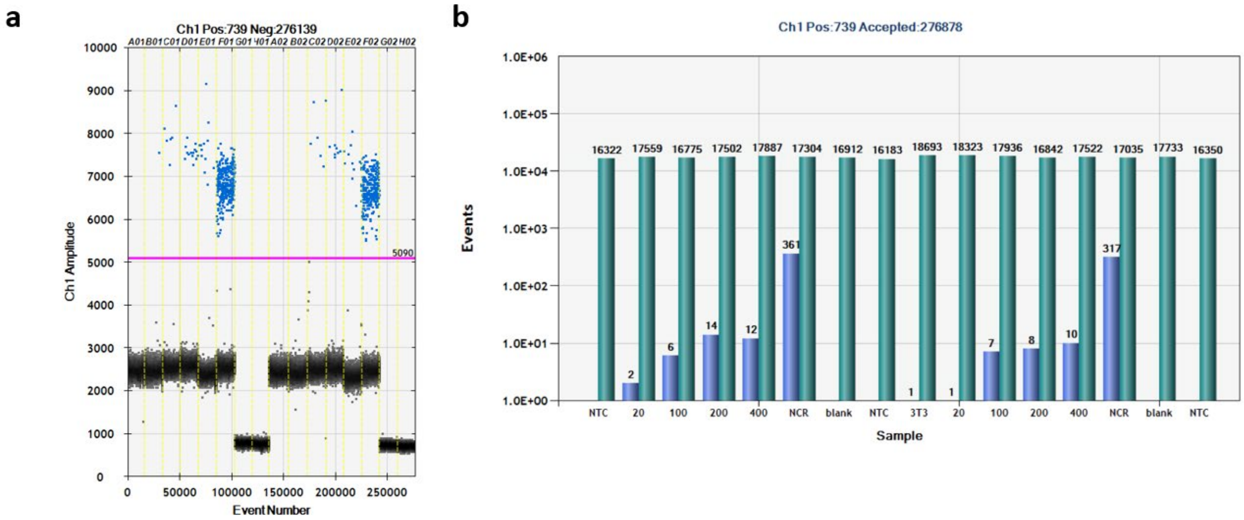

Supplemental Figure 4. Absolute event counts from ddPCR quantification of translocation efficiency. (a) The positive event threshold denoted by the pink line was set based on the positive control samples in lanes F01 and F02. (b) Absolute event counts from ddPCR. Each positive droplet is counted as a translocation event, shown in blue. Green bars represent the total number of droplets counted per sample.
